# An essential role for the intra-oocyte MAPK activity in the NSN-to-SN transition of germinal vesicle chromatin configuration in porcine oocytes

**DOI:** 10.1038/srep23555

**Published:** 2016-03-24

**Authors:** Ming-Ju Sun, Shuai Zhu, You-Wei Li, Juan Lin, Shuai Gong, Guang-Zhong Jiao, Fei Chen, Jing-He Tan

**Affiliations:** 1 College of Life Science, Northeast Agricultural University, Harbin, 150030, P.R. China; 2 College of Animal Science and Veterinary Medicine, Shandong Agricultural University, Tai-an City 271018, P.R. China

## Abstract

The mechanisms for the transition from non-surrounded nucleolus (NSN) to surrounded nucleolus (SN) chromatin configuration during oocyte growth/maturation are unclear. By manipulating enzyme activities and measuring important molecules using small-follicle pig oocytes with a high proportion of NSN configuration and an extended germinal vesicle stage *in vitro*, this study has the first time up-to-date established the essential role for intra-oocyte mitogen-activated protein kinase (MAPK) in the NSN-to-SN transition. Within the oocyte in 1–2 mm follicles, a cAMP decline activates MAPK, which prevents the NSN-to-SN transition by activating nuclear factor kappa-light-chain-enhancer of activated B cells (NF-κB) while inhibiting histone deacetylase (HDAC). In cumulus cells of 1–2 mm follicles, a lower level of estradiol and oocyte-derived paracrine factor (ODPF) reduces natriuretic peptide receptor 2 (NPR2) while enhancing FSH and cAMP actions. FSH elevates cAMP levels, which decreases NPR2 while activating MAPK. MAPK closes the gap junctions, which, together with the NPR2 decrease, reduces cyclic guanosine monophosphate (cGMP) delivery leading to the cAMP decline within oocytes. In 3–6 mm follicles, a higher level of estradiol and ODPF and a FSH shortage initiate a reversion of the above events leading to MAPK inactivation and NSN-to-SN transition within oocytes.

During their intra-follicular growth, oocytes synthesize and accumulate a large number of mRNAs and proteins required for meiotic maturation and early embryonic development. Concomitant with their increase in size, the nuclei (germinal vesicles or GV) of oocytes are subject to several levels of chromatin modifications and remodeling for the control of gene expression. These include not only the local chromatin modifications at specific promoter regions and cis-acting regulatory elements of single copy genes but also the large-scale chromatin remodeling throughout large areas of the genome^1^. This large-scale chromatin remodeling manifests as changes in GV chromatin configuration. For example, chromatin in growing mouse oocytes is initially decondensed in a non-surrounded nucleolus (NSN) configuration, but subsequently condensed, forming a surrounded nucleolus (SN) configuration with a heterochromatin rim around the nucleolus^2^,^3^,^4^,^5^. In pig oocytes, the diffuse chromatin (GV0) condenses successively into GV1, GV2, GV3 and GV4 conformations. While the GV0 is similar to the NSN in mouse oocytes, chromatin condensed into a ring or horseshoe around the nucleolus in all other patterns^6^,^7^,^8^.

Oocyte maturation includes both nuclear and cytoplasmic maturation. While nuclear maturation shows up as GV breakdown (GVBD) and extrusion of the first polar body, cytoplasmic maturation refers to acquisition of the competence to sustain maturation, fertilization and preimplantation development^9^,^10^,^11^. Generally speaking, cytoplasmic maturation is influenced primarily by epigenetic factors that control overall gene expression while inducing significant changes in GV chromatin configuration^1^,^12^,^13^,^14^. For example, oocytes must end a NSN configuration before gaining full competence to complete meiosis, and they must take on a SN configuration and stop gene transcription before being capable of sustaining blastocyst formation^12^. Thus, the NSN-to-SN transition is one of the most important aspects in oocyte cytoplasmic maturation. However, the mechanisms for the NSN-to-SN transition are not clear. Thus, the signaling pathways leading to the NSN-to-SN transition must be studied in order to better understand the epigenetic mechanisms of oocyte gene expression and to select oocytes that are more competent for *in vitro* maturation. Furthermore, such research will also provide new insights into the molecular regulation of somatic cell reprogramming.

Oocytes must be maintained at the GV stage in order to observe the NSN-to-SN transition *in vitro*. However, oocytes from many species quickly undergo GVBD after being released from follicles, and thus, GVBD must be inhibited with drugs to observe *in vitro* the NSN-to-SN transition in these species. Worse yet, drugs used for GVBD inhibition are often non-specific; they may affect the NSN-to-SN transition as well. Thus, oocytes with a naturally extended GV stage *in vitro* are ideal for studying NSN-to-SN transition. Previous studies indicated that porcine oocytes could maintain GV intact *in vitro* for an extended period of over 20 h^15^, and those from small follicles showed a high percentage (over 60%) of NSN configuration at the first release from follicles^8^. The aim of the present study was to explore the signaling pathways leading to oocyte NSN-to-SN transition by using pig oocytes from small antral follicles. The results have the first time up-to-date explored the signaling pathways leading to oocyte NSN-to-SN transition and established an essential role for intra-oocyte MAPK in the NSN-to-SN transition. The data not only will contribute to our understanding of the epigenetic mechanisms for oocyte maturation but also will provide important models for research on regulation of DNA transcription and the epigenetics and reprogramming in somatic cells.

## Results

### Classification of GV chromatin configuration and RNA transcription

**Figure 1:**
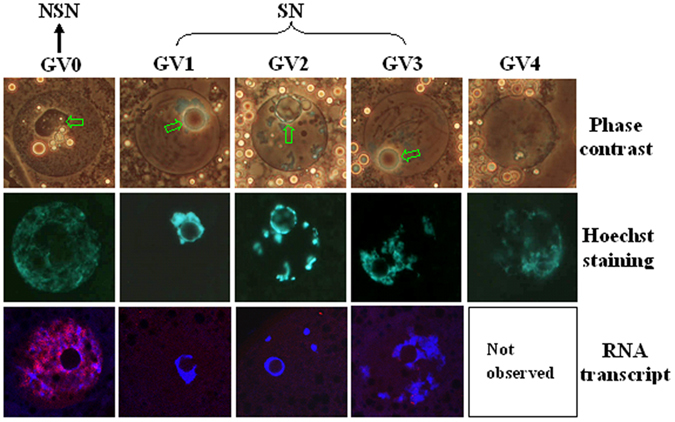
Photographs of porcine oocytes showing different germinal vesicle (GV) chromatin configurations and global RNA transcription. Photographs in the top and middle rows for each chromatin configuration are the same oocyte observed with phase contrast and fluorescence, respectively, after Hoechst 33342 staining. The nucleolus is indicated with arrows in the phase contrast images. Original magnification ×400. For convenience, GV-0 was designated as NSN configuration, and GV1, GV2 and GV3 were classed as SN configuration in the present study. Photographs in the bottom row are laser confocal (merged) images showing global RNA transcription of porcine oocytes with different GV chromatin configurations. DNA and RNA were pseudo colored blue and red, respectively. Original magnification ×630. Each treatment was repeated 3 times with each replicate containing about 30 oocytes.

The GV chromatin of porcine oocytes was classified into five configurations, based on the degree of chromatin condensation, and on disappearance of nucleolus and nuclear membrane (Fig. 1). The GV0 configuration was characterized by a distinct nucleolus and a diffuse, filamentous pattern of chromatin in the whole GV area. In GV1, the nucleolus was surrounded by a complete heterochromatin ring and heterochromatin was not obvious in the nucleoplasm. In GV2 and GV3, the heterochromatin ring around the nucleolus was often incomplete or forming a horseshoe, and clumps and strands of heterochromatin were observed in the GV. In GV4, the heterochromatin clumps or strands remained but the nuclear membrane was less distinct and the nucleolus disappeared completely. For convenience, GV0 was designated as NSN configuration, while GV1, GV2 and GV3 were classed as SN configuration in this study. Gene activities in oocytes with different chromatin configurations were determined by observing global RNA transcription after 5-ethynyl uridine (EU) labeling. Whereas the NSN (GV0) oocytes showed an intensive RNA transcription, no transcription was observed in GV1 and GV2 oocytes, and only faint labeling was observed in the GV3 oocytes (Fig. 1). Oocytes freshly collected from 1–2 mm follicles contained too few GV4 oocytes to observe RNA transcription.

### Role of MAPK in regulating the NSN-to-SN transition

**Figure 2:**
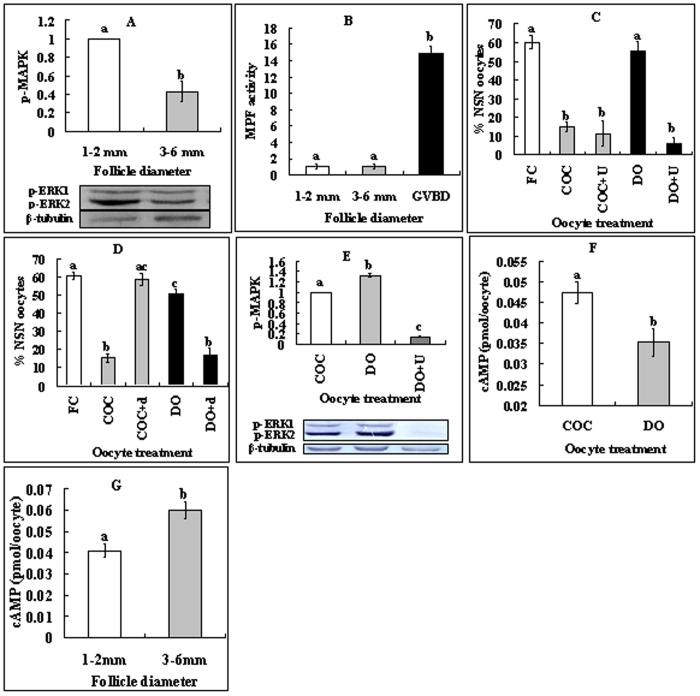
Roles of intra-oocyte MAPK, MPF and PKA in regulating the NSN-to-SN transition. (**A,B**) Levels of intra-oocyte p-MAPK and MPF activity, respectively, in oocytes from 1–2 mm or 3–6 mm follicles. Each treatment was repeated 3 times with each replicate containing 200 cumulus-free oocytes for p-MAPK and 40 oocytes for MPF activity assays. (**C,D**) Effects of inhibiting MAPK or activating PKA, respectively, on the NSN-to-SN transition. Freshly collected (FC) oocytes were cultured for 16 h as COCs or DOs in TCM-199 with (+) or without 20 μM U0126 (U) or 2 mM db-cAMP (d). Each treatment was repeated 4–5 times with each replicate containing about 25 oocytes. (**E**) Levels of intra-oocyte p-MAPK in oocytes cultured as COCs or DOs for 16 h with (+) or without U0126 (U). Each treatment was repeated 3 times with each replicate containing 200 cumulus-free oocytes. (**F**) Levels of intra-oocyte cAMP in oocytes cultured as COCs or DOs for 16 h without inhibitors. (**G**) Intra-oocyte cAMP levels in oocytes from follicles of different diameters. Each treatment was repeated 3 times with each replicate containing 100 cumulus-free oocytes. (a–c) Values without a common letter above their bars differ significantly (P < 0.05).

As MPF and MAPK are well-known molecules regulating GVBD, their roles in modulating NSN-SN transition were observed. Because around 60% of the oocytes from 1–2 mm follicles displayed NSN configurations while all the oocytes from 3–6 mm follicles had a SN configuration, the intra-oocyte MPF and MAPK activities were measured in these oocytes. The MAPK activity was significantly higher in oocytes from 1–2 mm follicles than in oocytes from 3–6 mm follicles (Fig. 2A). However, the MPF activity was hardly detectable in oocytes from either 1–2 or 3–6 mm follicles although it was obvious in GVBD oocytes (Fig. 2B). The results suggested that MAPK, but not MPF, was involved in regulating the NSN-to-SN transition.

To further study the role of MAPK in regulating the NSN-to-SN transition, COCs and DOs from 1–2 mm follicles were cultured with or without MAPK inhibitor, U0126. At 16 h of culture, over 90% of the oocytes showed intact GV with or without U0126. Whereas many NSN COCs had taken on the SN configuration without U0126, almost all the NSN DOs remained at the NSN stage (Fig. 2C). Culture with U0126 significantly facilitated the NSN-to-SN transition in DOs (Fig. 2C) while eliminating their MAPK activities almost completely (Fig. 2E). Furthermore, after culture without U0126, the level of intra-oocyte p-MAPK was significantly lower in COCs than in DOs (Fig. 2E). The results suggested that intra-oocyte MAPK inhibited the NSN-to-SN transition, whereas CCs facilitated the transition.

### Inactivation of protein kinase A (PKA) is required for activation of MAPK and inhibition of the NSN-to-SN transition

To answer the questions how the intra-oocyte MAPK activities are regulated and how CCs facilitate oocyte NSN-to-SN, effects of PKA activation on the NSN-to-SN transition were observed because PKA works upstream of MPF/MAPK^16^. COCs or DOs were cultured with or without PKA activator, db-cAMP. Although db-cAMP facilitated the NSN-to-SN transition of DOs as did MAPK inhibition, it inhibited NSN-to-SN in COCs (Fig. 2D) contrary to the effect of MAPK inhibition. After culture without db-cAMP, the level of intra-oocyte cAMP in COCs was significantly higher than that in DOs (Fig. 2F). Furthermore, oocytes from 1–2 mm follicles contained significantly less intra-oocyte cAMP than did the oocytes from 3–6 mm follicles (Fig. 2G). The results suggested that a lower level of intra-oocyte cAMP and hence inactivation of PKA activated MAPK, which inhibited the NSN-to-SN transition.

### MAPK prevents the NSN-to-SN transition by activating NF-κB (nuclear factor kappa-light-chain-enhancer of activated B cells) while inhibiting histone deacetylases (HDACs)

**Figure 3:**
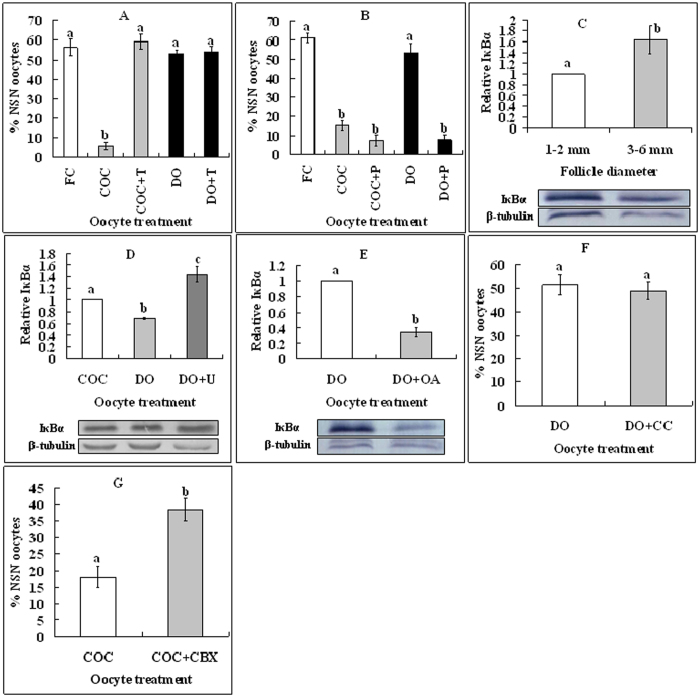
Roles of HDACs, NF-κB and GJC in regulating the NSN-to-SN transition. (**A,B**) Effects of inhibiting HDACs with TSA or inhibiting NF-κB with PDTC, respectively, on the NSN-to-SN transition. Freshly collected (FC) oocytes were cultured as COCs or DOs for 16 h in TCM-199 with (+) or without 400-nM TSA (T) or 50-μM PDTC (P). Each treatment was repeated 4–5 times with each replicate containing about 25 oocytes. (**C–E**) Levels of intra-oocyte IκBα (Western blotting). (**C**) Oocytes from 1–2 mm or 3–6 mm follicles, (**D**) After culture of COCs or DOs for 16 h with (+) or without U0126 (U), and (**E**) After culture of DOs with (+) or without OA. Each treatment was repeated 3 times with each replicate containing 200 cumulus-free oocytes. (**F**) Percentages of NSN oocytes after DOs were cultured alone or supplemented with (+) dispersed CCs. Each treatment was repeated 3–4 times with each replicate including 25 oocytes. (**G**) Percentages of NSN oocytes after COCs were cultured alone or supplemented with (+) CBX. Each treatment was repeated 4–5 times with each replicate including 25 oocytes. (a–c) Values without a common letter above their bars differ significantly (P < 0.05).

How did the intra-oocyte MAPK prevent NSN-to-SN? In somatic cells, the phosphorylation (activation) of nuclear NF-κB results in its association with CBP/p300 displacing HDAC-1 from DNA, leading to transcription activation^17^. MAPK can activate NF-κB through phosphorylation (inactivation) of inhibitory κBs (IκBs)^18^. We thus hypothesized that intra-oocyte MAPK might prevent NSN-to-SN by activating NF-κB. Roles of HDACs and NF-κB in regulating NSN-to-SN were therefore observed. COCs or DOs were cultured for 16 h with HDACs inhibitor, TSA or NF-κB inhibitor, PDTC. Whereas TSA inhibited NSN-to-SN in COCs (Fig. 3A), PDTC promoted it in DOs (Fig. 3B), suggesting that while HDACs promoted, NF-κB inhibited NSN-to-SN. Compared to oocytes from 1–2 mm follicles, oocytes from 3–6 mm follicles contained significantly more intra-oocyte IκBα (Fig. 3C) but less p-MAPK (Fig. 2A). After culture without inhibitors, the level of intra-oocyte IκBα was higher significantly in cultured COCs than in cultured DOs (Fig. 3D). Furthermore, whereas treating DOs with U0126 dramatically increased the expression of IκBα (Fig. 3D), treating DOs with OA to activate MAPK decreased IκBα significantly (Fig. 3E). Because all the oocytes from 3–6 mm follicles are of SN configuration, we propose that by inactivating IκB, intra-oocyte MAPK activates NF-κB, which prevents NSN-to-SN by displacing HDACs from DNA.

### Role of gap junction communications (GJC) in regulating the NSN-to-SN transition

Data in Fig. 2 indicated that whereas inhibiting MAPK facilitated, exposure to db-cAMP inhibited NSN-to-SN in COCs although db-cAMP facilitated NSN-to-SN in DOs. This suggested that db-cAMP had made CCs send signals that activated MAPK in the oocyte. In the LH receptor-activated signaling pathways regulating meiotic maturation, elevated levels of cAMP in CCs reduce cyclic guanosine monophosphate (cGMP) delivery to the oocyte and induce GVBD by down regulating NPR2 and blocking GJC via activating MAPK^19^. We thus hypothesized that elevated levels of cAMP in CCs would inhibit NSN-to-SN by reducing cGMP delivery to the oocyte. To test this hypothesis, role of GJC in regulating NSN-to-SN was first observed. DOs were cultured with or without dispersed CCs. No significant difference in the percentage NSN oocytes was observed between DOs cultured alone and DOs co-cultured with CCs (Fig. 3F). Effects of *in situ* blocking GJC on NSN-to-SN transition were then examined. COCs were cultured for 16 h with or without GJC blocker carbenoxolone (CBX) before chromatin configuration examination. Culture with CBX significantly inhibited the NSN-to-SN transition in COCs (Fig. 3G). The results confirmed that GJC were essential for CCs to facilitate the NSN-to-SN transition.

### Elevated cAMP levels activated MAPK while down regulating NPR2 in CCs

**Figure 4:**
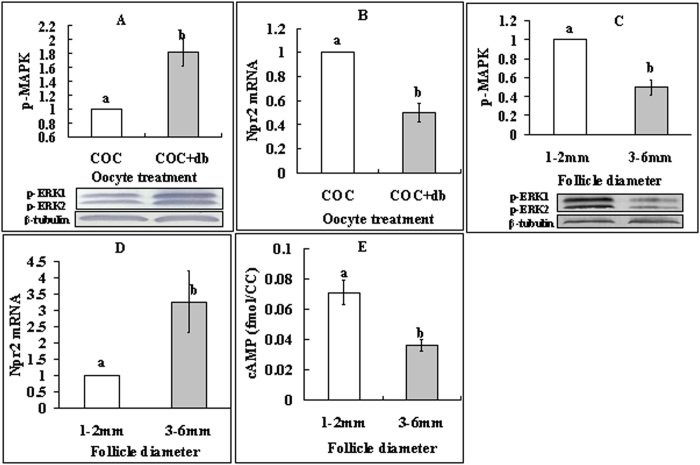
Levels of p-MAPK, Npr2 mRNA and cAMP in CCs. (**A,B**) show p-MAPK and Npr2 mRNA, respectively, in CCs from COCs that had been cultured for 16 h with (+db) or without db-cAMP. (**C–E**) show p-MAPK, Npr2 mRNA and cAMP, respectively, in CCs from follicles of different diameters. Each treatment was repeated 3 times with each replicate containing CCs from 200 COCs for p-MAPK and Npr2 mRNA assays and about 2 × 10^5^ CCs for cAMP measurement. (a,b) Values without a common letter above their bars differ significantly (P < 0.05).

To further confirm our hypothesis that cAMP in CCs inhibits NSN-to-SN by reducing cGMP delivery to the oocyte, effects of cAMP elevation on the activities of MAPK and NPR2 were observed in CCs. When COCs were cultured, the presence of db-cAMP increased the level of p-MAPK in CCs (Fig. 4A) while reducing their level of Npr2 mRNAs significantly (Fig. 4B). CCs from 1–2 mm follicles contained more p-MAPK (Fig. 4C) and cAMP (Fig. 4E) but less Npr2 mRNA (Fig. 4D) than did CCs from 3–6 mm follicles. The results suggested that, in CCs of small follicles, elevation of cAMP resulted in more p-MAPK but less NPR2, leading to a decrease in the cGMP delivery into the oocyte.

### FSH postponed the NSN-to-SN transition by activating MAPK while down regulating NPR2 in CCs

**Figure 5:**
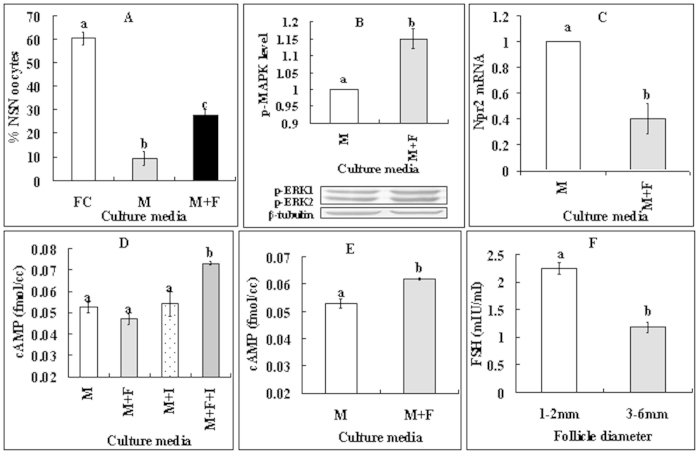
Effects of FSH on the NSN-to-SN transition. (**A–C**) Effects of FSH in culture media on NSN-to-SN of oocytes, and on levels of p-MAPK and Npr2 mRNA in CCs, respectively. (**D,E**) cAMP levels in CCs after culture of COCs or CCs, respectively. (**F**) FSH concentration in follicular fluid from 1–2 mm or 3–6 mm follicles. Freshly collected (FC) COCs were cultured for 14 h in TCM-199 (M) with (+) or without FSH (**F**) or ODPF inhibitors (I) before examination for chromatin configuration in oocytes or assay for p-MAPK, Npr2 mRNA or cAMP levels in CCs. To observe chromatin configuration, each treatment was repeated 4–5 times with each replicate containing about 25 oocytes. For p-MAPK and NPR2 mRNA assays in CCs, Each treatment was repeated 3 times with each replicate containing CCs from 200 COCs. For cAMP measurement in CCs, each treatment was repeated 3 times with each replicate including about 2 × 10^5^ CCs. For FSH assays in follicular fluid, each treatment was repeated 3 times with each replicate including 50 follicles. (a–c) Values without a common letter above their bars differ significantly (P < 0.05).

In the pig, FSH secretion is relatively constant throughout the estrous cycle^20^ and the growth of 1.1 to 2 mm follicles is FSH-dependent^21^. Furthermore, treatment with FSH stimulates cAMP accumulation in rat granulosa cells^22^. We thus proposed that FSH might inhibit NSN-to-SN by activating MAPK while down regulating NPR2 in CCs. We therefore observed the effects of FSH in culture media on NSN-to-SN in oocytes and on levels of p-MAPK, Npr2 mRNA and cAMP in CCs. COCs or CCs were cultured for 14 h with or without FSH supplementation. Percentages of the NSN oocytes were significantly higher in oocytes cultured with than without FSH supplementation (Fig. 5A). Whereas p-MAPK level increased (Fig. 5B), the level of Npr2 mRNA decreased (Fig. 5C) significantly in CCs in the presence of FSH. Although culture of COCs with FSH alone did not increase cAMP in CCs, culture of CCs with FSH alone or culture of COCs with both FSH and oocyte-derived paracrine factor (ODPF) inhibitors (SB431542 for GDF9 and LDN193189 for BMP15) significantly increased the cAMP level in CCs (Fig. 5D,E). Furthermore, follicular fluid from 1–2 mm follicles contained more FSH than did that from 3–6 mm follicles (Fig. 5F). The results (a) confirmed that FSH in culture media and in small follicles postponed NSN-to-SN by activating MAPK while down regulating NPR2 in CCs, and (b) suggested that ODPF inhibited FSH actions on cAMP production.

### ODPF and estradiol (E2) facilitated NSN-to-SN by enhancing CCs’ cGMP production and delivery to the oocyte

**Figure 6:**
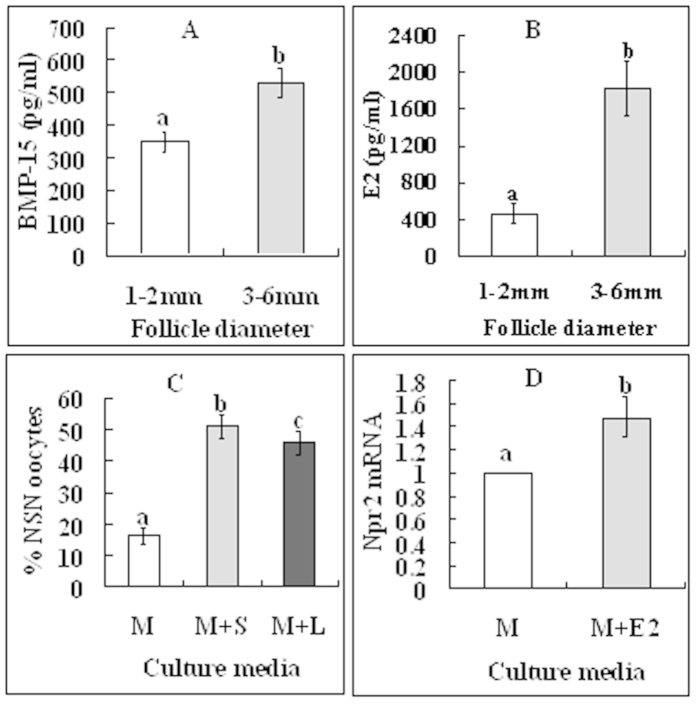
Roles of ODPF and estradiol in regulating the NSN-to-SN transition. (**A,B**) show concentrations of BMP-15 and E2, respectively, in follicular fluid from 1–2 mm and 3–6 mm follicles. Each treatment was repeated 3 times with each replicate containing follicular fluid from 50 follicles. (**C**) shows percentages of NSN oocytes after culture of COCs from 1–2 mm follicles in TCM-199 (M) with (+) or without ODPF inhibitors, SB431542 (S) for GDF9 or LDN193189 (L) for BMP15. Each treatment was repeated 3 times with each replicate containing 25 COCs. (**D**) shows relative levels of Npr2 mRNA in CCs after culture of COCs from 1–2 mm follicles in TCM-199 (M) with (+) or without E2. Each treatment was repeated 3 times with each replicate containing CCs from 200 COCs. (a–c) Values without a common letter above their bars differ significantly (P < 0.05).

There are reports that ODPF promotes Npr2 expression and elevates cGMP levels in CCs^23^, and that E2 is essential for the promotion and maintenance of NPR2 expression^24^. We thus hypothesize that 3–6 mm follicles would contain more ODPF and E2 in follicular fluid and more NPR2 in CCs than would 1–2 mm follicles, and that culture of COCs with ODPF inhibitors would inhibit NSN-to-SN and culture with E2 would increase NPR2 levels in CCs. Our measurement showed that the levels of BMP-15 and E2 in follicular fluid were significantly higher in 3–6 mm follicles than in 1–2 mm follicles (Fig. 6A,B). Figure 4D shows that CCs from 3–6 mm follicles contain more Npr2 mRNA than did those from 1–2 mm follicles. Furthermore, culture of COCs with ODPF inhibitors significantly inhibited oocyte NSN-to-SN (Fig. 6C) and culture with E2 increased NPR2 levels in CCs (Fig. 6D). The results suggested that ODPF and E2 facilitated NSN-to-SN by enhancing cGMP production and delivery to the oocyte.

## Discussion

**Figure 7:**
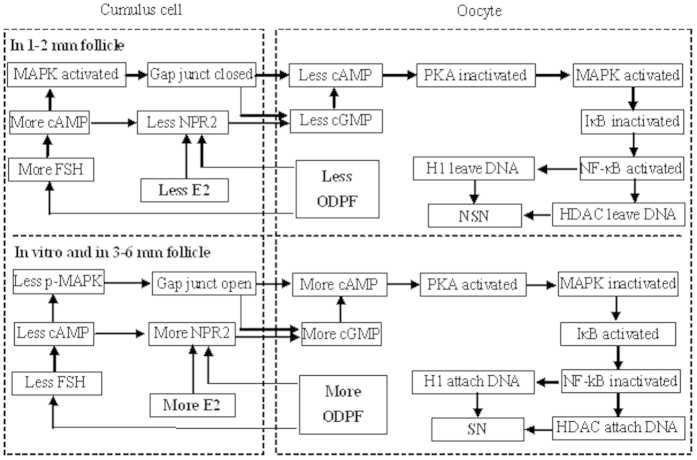
Possible signaling pathways leading to the NSN-to-SN transition in porcine oocytes. In CCs of 1–2 mm follicles, a lower level of ODPF and E2 reduces NPR2 while enhancing FSH and cAMP actions. FSH elevates the level of cAMP, which decreases NPR2 while activating MAPK. Activated MAPK closes the gap junction communications, which, together with the NPR2 decrease, reduces cGMP delivery leading to a cAMP decline within oocytes. Within the oocyte, the lower level of cAMP activates MAPK via inactivating PKA. MAPK activates NF-κB by inactivating IκB. Activated NF-κB displaces H1 and HDAC from DNA leading to chromatin decondensation (NSN). In 3–6 mm follicles, a significantly higher level of ODPF and E2 and a FSH shortage initiate a reversion of the above events leading to chromatin condensation (SN) in the oocyte.

By manipulating enzyme activities *in vitro* and by measuring related molecules both *in vivo* and *in vitro*, this study has established a pivotal role for MAPK in the regulation of the NSN-to-SN transition. Thus, the present results showed that the intra-oocyte MAPK inhibited GV chromatin condensation (the NSN-to-SN transition) of pig oocytes. Our further observation indicated that within the oocyte in small follicles, a lower level of cAMP activated MAPK, which activated NF-κB by inactivating IκB (Fig. 7). Activated NF-κB displaces H1 and HDAC from DNA, leading to chromatin decondensation (NSN). The roles of MAPK in follicle genesis/development, particularly those in meiosis progression, have been studied in several laboratories. For example, activated ERK 1/2 MAPK were localized in the cytoplasm of oocytes from gilts and sows, and their intensity did not differ among primordial/primary, secondary and tertiary follicles^25^. Although activation of MAPK in CCs is necessary for gonadotropin-induced GVBD of cultured COCs, MAPK activation is not required for spontaneous GVBD of cultured DOs^26^. Furthermore, by knocking out Erk1 and Erk2 in mouse oocytes, Zhang *et al.*^27^ observed that ERK1/2 activities in oocyte are dispensable for primordial follicle maintenance, activation and follicle growth. However, both the Mos null oocytes^28^ and the ERK1/2-deleted oocytes show anomalies during post-GVBD maturation and/or pronuclear formation, leading to subfertility or infertility. Taken together, the above data suggested that (1) for the first time, the current results demonstrated that compared to oocytes from medium-sized antral follicles, porcine oocytes from small follicles contained more activated MAPK, which inhibited the NSN-to-SN transition; (2) although the intra-oocyte MAPK activity is dispensable for GVBD, it plays an essential role in the NSN-to-SN transition; and (3) the abnormalities observed in the Mos null and ERK1/2-deleted mouse oocytes during post-GVBD maturation and pronuclear formation might suggest that due to a lack of inhibiting MAPK activities, a premature NSN-to-SN transition had occurred in these oocytes, which shut down the transcription of factors essential for oocyte final maturation and early embryo development.

It is well-known that MPF/MAPK facilitates post-GVBD chromosome condensation, but their role in GV chromatin condensation and DNA transcription has not been reported. Furthermore, the inhibitory effect of MAPK on GV chromatin condensation (NSN-to-SN transition) seems contradictory to their role in post-GVBD chromosome condensation. Studies on histone acetylation also indicated different mechanisms for the GV chromatin condensation and the post-GVBD chromosome condensation. For example, fully grown mouse oocytes (mostly of SN configuration) were fully acetylated at all the lysine residues on H3 and H4, but underwent deacetylation after GVBD^1^,^29^. Thus, the different mechanisms between the GV chromatin condensation and the post-GVBD chromosome condensation will be an very interesting topic in future studies.

It is known that NF-κB activation leads to expression of target genes by regulating chromatin structure^30^, and that NF-κB is maintained in a latent form in the cytoplasm by means of sequestration by IκB proteins. A significant increase of the IκBα-protein was observed as the NF-κB/p65-binding activity decreased with transcription silencing during the transition from fully-grown immature to *in vitro* matured MII bovine oocyte^31^. It was shown in somatic cells that MAPK could activate NF-κB through inactivating IκBs. For example, in human embryonic kidney 293 cells, over-expression of MEKK1 preferentially stimulates the kinase activity of IKKβ, which resulted in inactivation of IκBs^18^. Furthermore, MEKK1 stimulates the activities of both IKKα and IKKβ in transfected HeLa and COS-1 cells and directly phosphorylates the IKKs *in vitro*^32^. There are also numerous reports that NF-κB activation displaces histone H1 and HDAC from DNA, leading to chromatin relaxation. For example, activated nuclear NF-κB can bind to CBP/p300 and displace HDAC-1 from DNA^17^, and it was able to displace histone H1 and prevented its binding to nucleosome^33^. In addition, the NSN-to-SN transition was significantly impaired in the HDAC2 knockout (Hdac2^−/−^) mice^34^.

The present results indicated that in CCs of the small porcine follicles, a series of molecular events resulted in reduced production and delivery of cGMP into the oocyte, leading to a decline in intra-oocyte cAMP that inhibited the NSN-to-SN transition (Fig. 7). Firstly, the current results suggested that in small follicles, both a lower level of E2 and ODPF and a higher level of FSH inhibited the NSN-to-SN transition. According to Knox^35^, about 54% of the medium-sized (3–7 mm) porcine follicles are from ovaries at the early follicular, pro-estrous and estrous stages when large dominant follicles exist, but only about 44% of the small (<3 mm) follicles are from ovaries containing large dominant follicles. It is known that the dominant follicles produce a large quantity of estrogen, which turns down the pituitary secretion of FSH^36^. Thus, it was expected that most of the medium (subordinate) follicles would suffer FSH insufficiency and undergo atresia at this stage of follicle selection^37^. In both cows^38^ and sows^39^, the concentration of E2 increased significantly with increasing follicular sizes. Studies in the human suggested that GDF9 levels in the follicular fluid were highly correlated with oocyte nuclear maturation and embryo quality^40^. Porcine oocytes from <2 mm follicles showed significantly lower rates of maturation and blastocyst formation than oocytes from 3–6 mm follicles did after *in vitro* maturation^41^. Furthermore, a significant positive correlation was observed between BMP-15 and E2 levels in the same human follicle^42^.

Secondly, the present results suggested that in CCs of 1–2 mm follicles, FSH elevated cAMP levels, which activated MAPK while decreasing NPR2 expression (Fig. 7). There are reports that FSH stimulated cAMP accumulation during *in vitro* culture of rat granulosa cells^22^,^43^. LH-dependent activation of MAPK has been observed in granulosa cells of different species including the pig^44^. Further observations indicated that the LH-dependent MAPK activation occurred downstream of cAMP and was dependent on PKA activation^45^, and it might involve multiple signaling cascades downstream of the LH receptor, including the EGF receptor and possibly PKC pathways^46^. Likewise, the response of granulosa cells to FSH is also mediated by cAMP/PKA signaling and involves MAPK activation^45^,^47^. Furthermore, in their summary of the LH-induced EGFR trans-activation pathways leading to oocyte maturation, Conti *et al.*^19^ documented that cAMP inhibits cGMP production through the EGF network. In this study, culture of COCs with EGF postponed the NSN-to-SN transition to the same extent as did culture with FSH (data not shown). However, in the summary by Conti *et al.*^19^, whether cAMP can suppress NPR2 production directly is in question. Thus, the present results have provided the first direct evidence that cAMP inhibits cGMP production by decreasing NPR2 expression in CCs of small porcine follicles.

Thirdly, the current results suggested that in small porcine follicles, a lower level of ODPF and E2 inhibited the NSN-to-SN transition by reducing NPR2 expression and by removing the ODPF inhibition on FSH actions (Fig. 7). Furthermore, the activated MAPK prevented cGMP delivery into oocytes by closing GJC between CCs and the oocyte. It has been reported that ODPF, particularly the GDF-9-BMP-15 heterodimer, promotes Npr2 expression and elevates cGMP levels in CCs^23^. Whereas *in vitro* treatment of mouse COCs with FSH stimulated only a transient cAMP rise in CCs and oocytes, which disappeared after a 30 min culture^48^,^49^, FSH significantly increased cAMP production during a 48-h culture of rat granulosa cells^22^,^43^. There are reports that ODPF inhibits FSH actions with decreased cAMP production. For example, treatment of rat granulosa cells with GDF-9^43^, BMP-6^50^, or BMP-9^22^ significantly suppressed FSH-induced cAMP synthesis. Furthermore, BMP-15 inhibits FSH action by suppressing FSH receptor expression^51^. The ability of natriuretic peptide type C (NPPC) to keep meiotic arrest in cultured mouse COCs and the ability of CCs to produce cGMP were lost in the absence of E2, suggesting that E2 promotes and maintains expression of NPR2 in CCs and participates in the NPPC-mediated maintenance of oocyte meiotic arrest *in vitro*^24^. Furthermore, MAPK was found to mediate LH-induced oocyte maturation by interrupting GJC within the ovarian follicle through phosphorylation of connexin 43^52^,^53^.

In summary, the present results suggested that multiple factors including FSH, E2 and ODPF control the oocyte NSN-to-SN transition by acting on CCs, and that CCs regulate the transition by altering intra-oocyte cAMP levels via controlling cGMP production and delivery. Thus, in CCs of 1–2 mm follicles, a lower level of ODPF and E2 reduces NPR2 while enhancing FSH and cAMP actions (Fig. 7). FSH elevates the level of cAMP, which decreases NPR2 while activating MAPK. Activated MAPK closes the GJC, which, together with the NPR2 decrease, reduces cGMP delivery leading to a cAMP decline within the oocyte. Within the oocyte, the cAMP decline activates MAPK via inactivating PKA. By inactivating IκB, MAPK activates NF-κB, which displaces H1 and HDAC from DNA. As a result, oocytes remain at NSN. In 3–6 mm follicles, a significant increase in ODPF and E2 and a FSH shortage initiate a reversion of the above events leading to chromatin condensation (SN) in the oocyte. The results have the first time up-to-date explored the signaling pathways leading to oocyte NSN-to-SN transition in the mammalian species and have established an pivotal role for intra-oocyte MAPK in the regulation of the NSN-to-SN transition and hence DNA transcription. The data not only will contribute to our understanding of the epigenetic mechanisms for oocyte maturation but also will provide important models for research on the epigenetics and reprogramming in somatic cells.

## Methods

The experimental procedures used for animal care and handling were approved by the Animal Care and Use Committee of the Shandong Agricultural University P. R. China (Permit number: SDAUA-2001-001). The methods were carried out in accordance with the approved guidelines. Unless otherwise stated, all chemicals were obtained from Sigma-Aldrich Corp. (St. Louis, MO, USA).

### Recovery of oocytes

Porcine ovaries were collected at the Feicheng slaughterhouse of Yinbao Food Corporation Ltd. (Tai-an city, China) and transported to the laboratory within 3 h after slaughtering, in a thermos bottle with sterile saline containing 100 IU/ml penicillin and 0.05 mg/ml streptomycin, maintained at 30–35 °C. Cumulus-oocyte complexes (COCs) were recovered by aspirating 1–2 mm and 3–6 mm follicles using a syringe containing Dulbecco’s phosphate-buffered saline (D-PBS). Only COCs with uniform ooplasm and compact cumulus were chosen for further treatment.

### *In vitro* culture of oocytes

The basic culture medium (BCM) was TCM-199 (Gibco, Grand Island, New York, USA) supplemented with 0.91 mM sodium pyruvate, 1.0 g/L PVA, 3.05 mM D-glucose, 75 mg/ml penicillin G, 50 mg/ml streptomycin and 0.05 IU/ml FSH. To study the effect of FSH, FSH was removed from BCM. To study the effects of different signaling pathway regulators, BCM was supplemented with 20 μM U0126, 400 nM trichostatin A (TSA), 50 μM ammonium pyrrolidinedithiocarbamate (PDTC), 100 μM SB431542, 100 nM LDN193189, 100 μM Carbenoxolone (CBX), 1 μM okadaic acid (OA) or 2 mM db-cAMP. To make stock solutions, FSH (50 IU/ml), LDN193189 (100 μM), PDTC (100 mM) and CBX (100 mM) were dissolved in water, while U0126 (10 mM), db-cAMP (100 mM), SB431542 (20 mM), OA (200 μM) and TSA (5 mM) were dissolved in dimethyl sulfoxide (DMSO). All of the stock solutions were frozen stored in aliquots at −20 °C and diluted to the desired concentrations immediately before use.

Whereas some of the COCs were cultured directly after collection, others were mechanically denuded of cumulus cells (CCs) before culture as denuded oocytes (DOs). The COCs or DOs were cultured in groups of 20 in microdrops of 100 μl covered with mineral oil, at 38.5 °C under 5% CO_2_ in humidified air. To culture CCs, CCs released during oocyte denudation were washed twice in D-PBS by centrifugation (1500 × g, 5 min). Pellets were resuspended, and cells were counted on a hemocytometer. The final suspension (2–5 × 10^5^ cells/ml) was added to wells of 96-well culture plates (200 μl/well) and cultured at 38.5 °C in a humidified atmosphere of 5% CO_2_ in air.

### Observation of GV chromatin configuration and GVBD

Oocytes were denuded of CCs by pipetting in D-PBS containing 0.1% hyaluronidase. Oocytes were labeled for 10 min in D-PBS containing 10 mg/ml Hoechst 33342 at 38.5 °C under 5% CO_2_ in humidified air. Oocytes were then placed on glass slides and compressed with coverslips to visualize GV. The mounted oocytes were observed under a Leica DMLB microscope equipped with a CCD camera. Oocytes were first examined with phase contrast to visualize morphology of nucleoli and nuclear envelope, and then observed with fluorescence optics. Hoechst fluorescence was obtained by excitation at 220–360 nm using a mercury lamp (50 W) attenuated with neutral filters.

### Detection of global RNA transcription

Oocytes from 1–2 mm follicle were labeled for 2 h in 100 μl BCM containing 1 mM 5-ethynyl uridine (EU) at 38.5 °C under 5% CO_2_ in humidified air. All the steps of EU detection were performed at room temperature according to the manufacturer’s instructions (Invitrogen; Click-iT RNA imaging kits). After EU labeling, oocytes were (1) fixed using 3.7% formaldehyde in PBS for 40 min; (2) permeabilized with 0.1% Triton X-100 for 30 min; (3) stained for 30 min with 100 mM Tris (pH 8.5)/1 mM CuSO4/10–50 μM fluorescent azide/100 mM ascorbic acid, protected from light; (4) washed with Click-iT® reaction rinse buffer; (5) stained with Hoechst 33342; and (6) mounted on glass slides and observed with a Leica laser scanning confocal microscope (TCS SP2; Leica Microsystems). Blue diode (405 nm) and argon (Ar; 488 nm) lasers were used to excite Hoechst and FITC, respectively. Fluorescence was detected with the following bandpass emission filters: 420–480 nm for Hoechst and 505–540 nm for FITC.

### Western blot analysis

Two hundred cumulus-free oocytes or CCs from 200 COCs were frozen at −80 °C in a 1.5 ml microfuge tube containing 20 μl sample buffer (20 mM Hepes, 100 mM KCl, 5 mM MgCl_2_, 2 mM DTT, 0.3 mM PMSF, 3 mg/ml leupetin, pH 7.5). NaF (10 mM) was added to the sample buffer when phosphorylated mitogen-activated protein kinase (p-MAPK) was assayed. For protein extraction, 5 μl of 5 × SDS-PAGE loading buffer was added to each tube and the tubes were heated for 5 min at 100 °C. Total proteins were separated by SDS-PAGE on a 12% polyacrylamide gel and transferred onto PVDF membranes via electrophoresis. The membranes were then (1) washed twice in TBST (150 mM NaCl, 2 mM KCl, 25 mM Tris, 0.05% Tween-20, pH 7.4); (2) blocked for 1 h with 3% BSA at room temperature; (3) incubated at 4 °C overnight with primary antibodies (1:1000) in 3% BSA-TBST; (4) washed three times in TBST (5 min each) and incubated for 1 h at 37 °C with second antibodies (1:1000) in 3% BSA-TBST; (5) washed in TBST and detected by a BCIP/NBT alkaline phosphatase color development kit (Beyotime Institute of Biotechnology, China). The relative quantities of proteins were determined with an Image-Pro Plus software by analyzing the sum density of each protein band image. The quantity values of freshly collected GV oocytes were arbitrarily set as 100% and the other values were expressed relative to this activity. β-tubulin was assayed for internal controls. The primary antibodies used included rabbit anti-Phospho-p44/42 MAPK (Erk1/2) (Thr202/Tyr204) antibody (Cell Signaling, 9101), rabbit anti-IκB alpha antibody [E130] (abcam, ab32518), and mouse anti-β-tubulin (CWBIO, cw0098). The secondary antibodies included horse anti-mouse IgG AP conjugated (ZSGB-Biotechnology, ZB-2310) and goat anti-rabbit IgG AP conjugated (CWBIO, cw0111).

### *In vitro* p34^cdc2^ kinase (maturation-promoting factor, MPF) assay

The p34^cdc2^ kinase assay was performed using a MESACUP cdc2 kinase assay kit (code 5234; MBL, Nagoya, Japan) as described by Shoujo *et al.*^54^. Briefly, about 40 cumulus-free oocytes were placed in a plastic tube containing 10 μl of cell lysis buffer (50 mM Tris [pH 7.5], 150 mM NaCl, 2 mM EDTA, 5 mM EGTA, 1% [v/v] Triton X-100, 2.5 mM sodium pyrophosphate, 1 mM β-Glycerophosphate, 1 mM Na_3_VO_4_, 1 μg/ml of leupeptin, and 1 mM PMSF). Then, the tubes were frozen at −80 °C and thawed at room temperature three times. Cell extracts were frozen stored at −80 °C before use. Ten microliters of oocyte extracts were mixed with 35 μl of kinase assay buffer B (25 mM Hepes buffer [pH 7.5, MBL], 10 mM MgCl_2_ [MBL], 10% [v/v] MV peptide solution [SLYSSPGGAYC; MBL], 0.1 mM ATP), and the mixture was incubated for 30 min at 30 °C. The reaction was terminated by the addition of 200 ml PBS containing 50 mM EGTA (MBL). The phosphorylation of MV peptides was detected at 492 nm using a plate reader (BioTek-ELx808, BioTek Instruments, Inc.). Data were expressed as the fold strength of p34^cdc2^ kinase activity in oocytes from 1–2 mm follicles.

### Real time PCR

CCs from 200 COCs were treated with TRIzol reagent for RNA isolation. The RNA isolated was resuspended in diethyl pyrocar-bonate-treated MilliQ water (DEPC-dH_2_O) and digested with RNase-free DNase I (Takara Biotechniques). The purified RNA was dissolved in DEPC-dH_2_O and spectroscopically quantified at 260 nm. Purity and integrity of the RNA was assessed by determination of the A_260_:A_280_ ratio (1.8–2.0) and electrophoresis in 1% agarose.

Reverse transcription was performed in a total volume of 20 μl using Superscript III Reverse Transcriptase (Invitrogen Australia Pty., Ltd). Briefly, 2 μl RNA sample were mixed in a 0.2-ml reaction tube with 4 μl dNTP, 1.5 μl Oligo dT18 (Takara) and 6 μl DEPC-dH_2_O, and the mixture was incubated in a PCR instrument at 65 °C for 5 min. Soon after the incubation, the reaction tube was cooled on ice for 2 min and centrifuged (200 × g for 10 sec at 4 °C). Then, 4 μl 5 × RT buffer, 0.5 μl RNase inhibitor and 0.5 μl Superscript III Reverse Transcriptase were added to the reaction tube. The mixture was incubated at 50 °C for 1 h, and then, at 70 °C for 15 min before storage at −20 °C until use.

Amplification was carried out using designed primers of porcine natriuretic peptide receptor 2 (NPR2) (forward 5′-CTGCTTCCCGAATGGAGTCTA-3′, reverse 5′-TAGGAGCCAGTAAGTT CGCATC-3′) and a β-actin gene as control (forward 5′-CGTGCGGGACATCAAGGA-3′, reverse 5′-AGGAAGGAGGGCTGGAAGA-3′). Quantification of mRNA was conducted using the Mx3005P Real-Time PCR System (Stratagene, Valencia, CA). Amplification reactions were performed in a 10 μl reaction volume containing 1 μl cDNA, 5 μl 2 × SYBR Green Master Mix (Stratagene), 0.15 μl 500-fold diluted reference dye, 3.05 μl RNase-free water, and 0.4 μl each of forward and reverse gene-specific primers (10 μM). Cycle amplification conditions included an initial denaturation at 95 °C for 10 min, 40 cycles at 95 °C for 5 sec and 60 °C for 20 sec. Immediately after amplification, PCR products were analyzed by sequencing, dissociation-curve analysis and gel electrophoresis to determine specificity of the reaction. Gene expression was normalized to the β-actin internal control. All values were then expressed relative to calibrator samples using the 2^−(ΔΔCT)^ method^55^.

### Enzyme-linked immunosorbent assay (ELISA)

Two hundred and fifty cumulus-free oocytes or 2 × 10^5^ CCs from COCs were placed in a freezing deposit tube containing 100 μl PBS and frozen in liquid nitrogen. The tubes were then frozen-thawed three times and the cell extracts isolated were frozen stored at −80 °C until use. Follicular fluid was centrifuged for 10 min at 3000 × g at 4 °C and stored at −80 °C until use. Concentrations of cAMP were measured using a porcine cAMP Elisa kit (BLUE GENE, Shanghai, China) and BMP-15 was measured using a porcine BMP-15 Elisa kit (BLUE GENE, Shanghai, China). Briefly, standards or samples were added in duplicate to wells of a micro-titer plate pre-coated with porcine cAMP or BMP-15 monoclonal antibodies, then 50 μl Conjugate was added to each well and incubated for 1 h at 37 °C. After color reaction the optical density was measured at 450 nm using a plate reader (BioTek-ELx808, BioTek Instruments, Inc.). The concentrations of cAMP or BMP-15 were calculated according to their repective standard curves.

### FSH and estradiol (E2) measurement

Radioimmunoassay for estradiol and FSH were conducted by the Central Hospital of Tai-An City using commercial RIA kits from Beifang Biomedical Techniques Co. Ltd., Beijing, China and XieHe medical Techniques Co. Ltd., Tianjin, China, respectively. Briefly, the follicular fluid collected from different sizes of follicles were centrifuged for 10 min at 3000 × g at 4 °C and stored at −80 °C until use. The minimum detectable amounts of estradiol and FSH were 1.4 pg/ml and 0.46 mIU/ml, respectively. The intra- and inter-assay coefficients of variation were all <10% and <15%.

### Data analysis

We conducted at least three replicate trials for each treatment. Data were analyzed by ANOVA, using SPSS (Statistics Package for Social Science) software. Data were compared using one-way analysis of variance after being transformed via LSD and P < 0.05 was considered significant.

## Additional Information

**How to cite this article**: Sun, M.-J. *et al.* An essential role for the intra-oocyte MAPK activity in the NSN-to-SN transition of germinal vesicle chromatin configuration in porcine oocytes. *Sci. Rep.* **6**, 23555; doi: 10.1038/srep23555 (2016).
